# Risk factors of chronic periodontitis on healing response: a multilevel modelling analysis

**DOI:** 10.1186/s12911-017-0533-2

**Published:** 2017-09-15

**Authors:** J. Song, H. Zhao, C. Pan, C. Li, J. Liu, Y. Pan

**Affiliations:** Department of Periodontics and Oral Biology, School of Stomatology, China Medical University, Shenyang, 110002 China

**Keywords:** Chronic periodontitis, Risk factors, Prognosis, Multilevel modelling

## Abstract

**Background:**

Chronic periodontitis is a multifactorial polygenetic disease with an increasing number of associated factors that have been identified over recent decades. Longitudinal epidemiologic studies have demonstrated that the risk factors were related to the progression of the disease. A traditional multivariate regression model was used to find risk factors associated with chronic periodontitis. However, the approach requirement of standard statistical procedures demands individual independence. Multilevel modelling (MLM) data analysis has widely been used in recent years, regarding thorough hierarchical structuring of the data, decomposing the error terms into different levels, and providing a new analytic method and framework for solving this problem. The purpose of our study is to investigate the relationship of clinical periodontal index and the risk factors in chronic periodontitis through MLM analysis and to identify high-risk individuals in the clinical setting.

**Methods:**

Fifty-four patients with moderate to severe periodontitis were included. They were treated by means of non-surgical periodontal therapy, and then made follow-up visits regularly at 3, 6, and 12 months after therapy. Each patient answered a questionnaire survey and underwent measurement of clinical periodontal parameters.

**Results:**

Compared with baseline, probing depth (PD) and clinical attachment loss (CAL) improved significantly after non-surgical periodontal therapy with regular follow-up visits at 3, 6, and 12 months after therapy. The null model and variance component models with no independent variables included were initially obtained to investigate the variance of the PD and CAL reductions across all three levels, and they showed a statistically significant difference (*P < 0.001*), thus establishing that MLM data analysis was necessary. Site-level had effects on PD and CAL reduction; those variables could explain 77–78% of PD reduction and 70–80% of CAL reduction at 3, 6, and 12 months. Other levels only explain 20–30% of PD and CAL reductions. Site-level had the greatest effect on PD and CAL reduction.

**Conclusions:**

Non-surgical periodontal therapy with regular follow-up visits had a remarkable curative effect. All three levels had a substantial influence on the reduction of PD and CAL. Site-level had the largest effect on PD and CAL reductions.

**Electronic supplementary material:**

The online version of this article (10.1186/s12911-017-0533-2) contains supplementary material, which is available to authorized users.

## Background

Chronic periodontitis is a multifactorial polygenetic disease with an increasing number of associated factors that have been identified over recent decades. Longitudinal epidemiologic studies have demonstrated that the risk factors were related to the progression of the disease [1, 2]. Plaque is an initial and necessary factor for the development of periodontal disease, but it alone does not necessitate periodontitis. The combined effects of different risk factors associated with periodontitis will eventually lead to periodontitis. Currently, some known risk factors associated with periodontitis include plaque, bleeding on probing (BOP), tooth position, age, gender, socio-economic position, smoking, stress-experience, obesity, systemic disease, and more [3–6].^.^ In the past 30 years, one of the great advances in periodontology was the discovery of a huge difference in peoples’ susceptibility to periodontitis, which was mainly demonstrated in the occurrence, development, and response to treatment. Risk factor assessment and control have been paid increasing amounts of attention due to the belief that these are important parts of periodontal diagnosis and treatment [7]. Recently, researchers have proposed periodontal risk assessments (PRA) or periodontal risk calculators (PRC) [8, 9]. The basic idea is to put various periodontal risk factors together for analysis to help doctors more accurately analyse the progression and prognosis of diseases and then plan customized treatment for each individual.

A traditional multivariate regression model was used to find risk factors associated with chronic periodontitis. However, the approach requirement of standard statistical procedures demands individual independence. If this is not done, the analysis will give incorrect or potentially misleading results. Another drawback is that in averaging lower level data to a higher level, the data is artificially simplified; consequently, the statistical power will appear biased. Multilevel modelling (MLM) data analysis has widely been used in recent years, regarding thorough hierarchical structuring of the data, decomposing the error terms into different levels, and providing a new analytic method and framework for solving this problem [10].

In much of periodontal research, statistical methods assume that the site observations are independent and use the mean values of sites to represent the full mouth. Periodontal data with an inherently hierarchical structure poses difficulties for analysis, as sites are clustered around a tooth, and teeth are clustered within the patient. The statistical approach of MLM may solve this problem. MLM has been widely used in education research, management, economics, sociology, psychology, and other fields but less for medical research [11]. In 1992, Albandar [12] stated that the application of multilevel modelling analysis on periodontal research may have important significance. Application of multilevel modelling analysis may provide a more accurate explanation of the natural hierarchical structure of the progression and healing responses after periodontal treatment [13].

MLM can provide a novel approach for periodontal disease research [14, 15]. The aim of the present study was to investigate, by means of multilevel analysis, risk factors that may affect moderate to severe periodontitis prognosis in Northern Chinese who have undergone non-surgical periodontal treatment.

## Methods

### Selection of patients

The study sample comprised 54 patients who had been referred to the Department of Periodontics at the School of Stomatology, China Medical University, during the years 2012 through 2014. Based on sample size estimation of repeated measurement design, we calculated the statistical power using Stata 10.0. Statistical power was close to 1 in our study. Our statistical power was high, therefore patient number was sufficient in our study. The patients had presented with moderate to severe periodontitis ((i) loss of at least 10 teeth, i.e., ≤ 18 existing teeth; or (ii) at least two interproximal sites (not on same tooth) with ≥6 mm clinical attachment loss and at least one interproximal site with ≥5 mm pocket depth; or (iii) at least two interproximal sites with 4–5 mm clinical attachment loss and no interproximal sites with clinical attachment loss of ≥6 mm, and the percentage of sites with pocket depth ≥ 4 mm in the upper tertile of the study population) [16]. All the patients received non-surgical periodontal treatments well as follow-up visits at 3, 6 and 12 months.

Patients were included if they displayed the following features:

1. Were 35 to 60-years-old and from northeast China, with no prior periodontal or orthodontic treatment.
2. Had at least 16 standing teeth (excluding the third molars).
3. Had no serious mental illness or cognitive dysfunction.

Patients were excluded if the patient interview revealed any of the following:

1. Known systemic diseases.
2. Patient did not consent to non-surgical periodontal therapy or regular follow-up visits.
3. A history of systemic antibiotic or periodontal treatment in the preceding 3 months.
4. Pregnant or lactating females.
5. Smoking cessation.

Eligible patients who agreed to participate were asked to complete an in-person verbally administered survey. Written consent was obtained prior to the study participation. To ensure that each participant, regardless of his or her literacy level, understood and was willing to participate in the study, the consent form was read to all participants and they consented to the collection of data for the analysis of clinical research.

### Clinical measurements

To obtain personal information, a questionnaire that included Gender(Male/Female), Age, Stress-experience(yes/no), BMI, Education background(post graduation / graduate / senior school or below), Smokers, Socio-economic (More than 599.60 USD = 1, 299.76–599.60 USD =2, 149.88–299.76 USD = 3, 74.94–149.88 USD =4, less than 74.94 USD = 5) was distributed among all the patients. Additional file 1. All patients received the same non-surgical periodontal treatment, including oral hygiene instruction regarding brushing and inter-dental cleaning, and then regular follow-up visits at 3, 6, and 12 months after treatment. This was a 12-month prospective study. The data collection was conducted at baseline and 3, 6, and 12 months after completion of non-surgical periodontal treatment. The clinical examination and non-surgical periodontal treatment of all patients was performed by a calibrated examiner from the periodontic department.

Four sites (mesio-buccal, mid-buccal, disto-buccal, and mid-lingual) per tooth were measured and recorded, excluding the third molars. PD, CAL, BOP, presence of plaque, mobility, and the number of missing teeth were probed with an automated controlled force periodontal probe (Florida Probe 32, Version 6, USA).

### Data analysis

All data were entered in EpiData 3.1 and proofed for entry errors. SPSS12.0 and HLM6.0 statistical software were used to analyse the data.

MLM data analysis was used to analyse PD and CAL reductions at the site level at 3, 6, and 12 months compared with baseline PD and CAL. The hierarchical analysis was constructed on three levels: the patient, the tooth, and the tooth site. The patient-level had 11 variables, including gender, age, smoker, BMI, stress-experience, socio-economic position, education level, the number of missing teeth, percentage sites with plaque at baseline, percentage sites with BOP at baseline, and percentage sites with PD ≥ 5 mm at baseline. The tooth-level had 2 variables, including tooth position and mobility. The site-level had 3 variables, including plaque at baseline, BOP at baseline, and surface of tooth. The total number of variables associated with all the three levels are shown in Table 1.

**Table 1:** Variable assignment table

Variables	Assignment
Patient -level	Level 3
Age	continuous variable
Gender	female =1, male =2
Smokers	yes = 1, no = 0
BMI	continuous variable
Socio-economic	More than 599.60 USD = 1, 299.76–599.60 USD =2, 149.88–299.76 USD = 3, 74.94–149.88 USD =4, less than 74.94 USD = 5
Education background	post graduation =1, graduate =2, senior school or below =3
Stress-experience	yes = 1,no = 0
Number of missing teeth	continuous variable
% of sites with plaque at baseline	continuous variable
% of sites with BOP at baseline	continuous variable
% of sites with PD ≥ 5 mm at baseline	continuous variable
Tooth-level	Level 2
Tooth position	anterior = 1, premolar = 2, molar = 3
Mobility	Do not have mobility = 0, mobility ≤ 1 mm = 1, 1 mm < mobility ≤ 2 mm = 2, mobility > 2 mm = 3
Site-level	Level 1
Plaque at baseline	presence = 1, absence = 0
BOP at baseline	presence = 1, absence = 0
Surface of tooth	1 = buccal / lingual, 2 = mesiocclusion/distocclusion

## Results

In this study, 54 patients were investigated. A summary of each level and variable is described in Table 2. The mean age was 44.7 ± 1 years old, ranging from 35 to 59, and the sample was equally distributed in terms of gender (55.6% females). Fourteen patients were smokers among the males, and there were no female smokers. The mean BMI was 23.2 ± 0.5 kg/m^2^. There were 5 patients who socio-economic position in terms of income monthly was more than 599.60 USD, 28 patients with 299.76–599.60 USD, 13 patients with 149.88–299.76 USD, 6 patients with 74.94–149.88, and 2 patients with less than 74.94 USD. There were 5 patients with a post-graduate education level, 38 patients with a graduate level, and 11 patients with senior school or below. 57.4% (31/54) of the patients had stress-experience. The mean number of missing teeth was 1.5 ± 0.2. The sample included a total of 1431 teeth, included 641 anterior teeth, 413 premolars, and 377 M. There were 1131 teeth with mobility of 0, 202 teeth with mobility of 1, 80 teeth with mobility of 2, and 18 teeth with mobility of 3. The mean percentage sites with plaque at baseline were79.3% ± 1.6%. The mean percentage sites with BOP at baseline were 66.5% ± 2.0%. The mean percentage sites with PD ≥ 5 mm at baseline were 28.3% ± 1.8%. Sites with positive plaque index totaled 4547, while 3784 sites were positive in BOP index, making a total of 5724 sites at baseline.

**Table 2:** Baseline clinical and periodontal parameters by variables

Variable	Mean ± SD
Patient -level (*N* = 54)	
Age (years)	44.7 ± 1
BMI(kg/ m^2^)	23.2 ± 0.5
Number of missing teeth (N)	1.5 ± 0.2
% of sites with plaque at baseline	1.11 ± 0.15^a^
% of sites with BOP at baseline	0.97 ± 0.17 ^a^
% of sites with PD ≥ 5 mm at baseline	0.55 ± 0.15 ^a^
Variable	N (%)
Patient-level (N = 54)	
Gender	
Male	24(44.4%)
Female	30(55.6%)
Smokers	
Yes	14(25.9%)
No	40(74.1%)
Socio-economic	
More than 599.60 USD	5 (9.3%)
299.76–599.60 USD	28 (51.8%)
149.88–299.76 USD	13 (24.1)
74.94–149.88 USD	6 (11.1%)
Less than 74.94 USD = 5	2 (3.7%)
Education background (N)	
post graduation	5 (9.3%)
Graduate	38 (70.4%)
senior school or below	11(20.3%)
Stress-experience	
Yes	31(57.4%)
No	23(42.6%)
Tooth-level (*N* = 1431)	
Tooth position	
Anterior	641 (44.8%)
Premolar	413 (28.9%)
Molar	377 (26.3%)
Mobility	
Do not have mobility	1131 (79.0%)
mobility ≤ 1 mm	202 (14.1%)
1 mm < mobility ≤ 2 mm	80 (5.6%)
mobility > 2 mm	18 (1.3%)
Site-level (*N* = 5724)	
Plaque at baseline	
Presence	4547(79.4%)
Absence	1177(20.6%)
BOP at baseline	
Presence	3784(66.1%)
Absence	1940(33.9%)
Surface of tooth	
buccal / lingual	2862(50%)
mesiocclusion/distocclusion	2862(50%)

a: data after arcsine square root transformation

In response to non-surgical periodontal treatment, the full-mouth mean PD and CAL showed significant reductions at 3, 6, and 12 months compared with the baseline. The mean PD was 3.83 ± 0.17 mm at baseline, 2.04 ± 0.01 mm at 3 months, 2.01 ± 0.01 mm at 6 months, 1.19 ± 0.01 mm at 12 months. The mean CAL was 3.70 ± 0.03 mm at baseline, 3.04 ± 0.02 mm at 3 months, 2.98 ± 0.02 mm at 6 months, 2.89 ± 0.02 mm at 12 months.

### Multilevel statistical analysis

Traditional statistical methods require individual independent variables, but the site-level observations in this study are not truly independent. Consequently, MLM analysis was used to investigate the possible factors in response to non-surgical periodontal treatment in terms of both PD and CAL reduction.

### Diversity of PD and CAL reductions between teeth

The null model was the basis upon which to use MLM analysis. When different levels were determined to be correlated, MLM analysis was used; otherwise, a traditional statistical method was used. To investigate the reduction of PD and CAL across all three levels, the null model with the independent variables included were established (Table 3).

$$ \mathrm{Level}\hbox{-} 1:{\mathrm{Y}}_{\mathrm{ijk}}={\upbeta}_{\mathrm{ojk}}+{\upgamma}_{\mathrm{ijk}} $$

$$ \mathrm{Level}\hbox{-} 2:{\upbeta}_{\mathrm{ojk}}={\upgamma}_{00\mathrm{k}}+{\upmu}_{\mathrm{ojk}} $$

$$ \mathrm{Level}\hbox{-} 3:{\upgamma}_{00\mathrm{k}}={\uppi}_{000}+{\mathrm{e}}_{\mathrm{ook}} $$

**Table 3:** Variance Components models for reduction in PD and CAL

Variance	SE	Variance Components	*p*
The reduction in 3-month PD			
Patient (level-3)	0.40	0.16 (10%)	504.21^***^
Tooth (level-2)	0.44	0.20(12%)	2230.98^***^
Site (level-1)	1.13	1.26(78%)	
The reduction in 6-month PD			
Patient (level-3)	0.44	0.19(12%)	624.64^***^
Tooth (level-2)	0.42	0.18(11%)	2146.62^***^
Site (level-1)	1.12	1.25(77%)	
The reduction in 12-month PD			
Patient (level-3)	0.45	0.20(11%)	566.97^***^
Tooth (level-2)	0.45	0.20(11%)	2173.27^***^
Site (level-1)	1.19	1.41(78%)	
The reduction in 3-month CAL			
Patient (level-3)	0.72	0.52(16%)	863.59^***^
Tooth (level-2)	0.59	0.35(11%)	2217.20^***^
Site (level-1)	1.52	2.30(73%)	
The reduction in 6-month CAL			
Patient (level-3)	0.64	0.40(14%)	748.89^***^
Tooth (level-2)	0.51	0.26(10%)	2019.35^***^
Site (level-1)	1.50	2.26(76%)	
The reduction in 12-month CAL			
Patient (level-3)	0.63	0.40(13%)	700.51^***^
Tooth (level-2)	0.56	0.31(10%)	2118.56^***^
Site (level-1)	1.51	2.29(77%)	

^***^ *p<0.001*

Y_ijk_, indicates the reduction in PD and CAL in patient Y, with “i” as the patient number, “j” as the tooth number, and “k”as the site number. Β_ojk_ was the mean reduction of patient Y, γ_00k_was the mean reduction of tooth β_ojk_, and π_000_ was the mean reduction of site γ_00k_. γ_ijk_ represented the variation between the sites, which was the random component of Y_ijk_. μ_ojk_ represented the variation between the teeth, which was the random component of β_ojk_. E_ook_ represented the variation between the patients, which was the random component of γ_00k_.

The variance components showed that significant variations existed in all three levels *(p < 0.001*).Thus, it was necessary to use MLM analysis. The reduction of PD and CAL was significantly different among the teeth. Site-level significantly affected PD and CAL reductions: the variables could explain 77–78% of PD reduction and 70–80% of CAL reduction at 3, 6, and 12 months. As for the tooth-level, the variables could explain 11–12% of PD reduction and 10–11% of CAL reduction at 3, 6, and 12 months. For patient-level effect on PD and CAL reductions, the variance explained 10–11% of PD reduction and 13–14% of CAL reduction at 3, 6, and 12 months.

### The influence of variables on PD and CAL reduction

A total of 16 independent variables, with 11 on the patient level, 2 on the tooth level, and 3 on the site level, were included in the MLM analysis.

Level-1:

$$ {\mathrm{Y}}_{\mathrm{ijk}}={\upbeta}_{\mathrm{ojk}}+{\upbeta}_{1\mathrm{jk}}{\mathrm{X}}_{1\mathrm{ijk}}+{\upbeta}_{2\mathrm{jk}}{\mathrm{X}}_{2\mathrm{ijk}}+{\upbeta}_{3\mathrm{jk}}{\mathrm{X}}_{3\mathrm{ijk}}+{\upgamma}_{\mathrm{ijk}} $$

Level-2:

$$ {\upbeta}_{\mathrm{ojk}}={\upgamma}_{00\mathrm{k}}+{\upgamma}_{01\mathrm{k}}{\mathrm{W}}_{1\mathrm{jk}}+{\upgamma}_{02\mathrm{k}}{\mathrm{W}}_{2\mathrm{jk}}+{\upmu}_{\mathrm{ojk}} $$

$$ {\upbeta}_{1\mathrm{jk}}={\upgamma}_{10\mathrm{k}} $$

$$ {\upbeta}_{2\mathrm{jk}}={\upgamma}_{20\mathrm{k}} $$

$$ {\upbeta}_{3\mathrm{jk}}={\upgamma}_{30\mathrm{k}} $$

Level-3:

$$ {\displaystyle \begin{array}{l}{\upgamma}_{00\mathrm{k}}={\uppi}_{000}+{\uppi}_{001}{\mathrm{Z}}_{00\mathrm{k}}+{\uppi}_{002}{\mathrm{Z}}_{01\mathrm{k}}+{\uppi}_{003}{\mathrm{Z}}_{03\mathrm{k}}+{\uppi}_{004}{\mathrm{Z}}_{04\mathrm{k}}+{\uppi}_{005}{\mathrm{Z}}_{05\mathrm{k}}+{\uppi}_{006}{\mathrm{Z}}_{06\mathrm{k}}+{\uppi}_{007}{\mathrm{Z}}_{07\mathrm{k}}+{\uppi}_{008}{\mathrm{Z}}_{08\mathrm{k}}+\\ {}{\uppi}_{009}{\mathrm{Z}}_{09\mathrm{k}}+{\uppi}_{010}{\mathrm{Z}}_{10\mathrm{k}}+{\uppi}_{011}{\mathrm{Z}}_{11\mathrm{k}}+{\mathrm{e}}_{\mathrm{ook}}\end{array}} $$

$$ {\upgamma}_{01\mathrm{k}}={\uppi}_{010} $$

$$ {\upgamma}_{02\mathrm{k}}={\uppi}_{020} $$

The relationship of the risk factors and PD on healing response.

A total of 5724 sites distributed on 1431 teeth in the 54 patients were included in this study. Compared with the baseline, the mean reduction in PD was 1.79 mm, 1.82 mm, 2.64 mm at 3, 6, and 12 months.

At the site level, presence of BOP at baseline and the mesiocclusion/distocclusion surface of teeth showed a significantly greater reduction in PD at 3, 6, and 12 months (*p < 0.05*). The variables on the site level were reduced by 7%, 6%, 7% of PD reduction at 3, 6, and 12 months when compared with the corresponding Variance Component Models (Table 4).

**Table 4:** Random intercept models for reduction in PD and CAL

	PD			CAL		
	3-month Estimate (SE)	6-month Estimate (SE)	12-month Estimate (SE)	3-month Estimate (SE)	6-month Estimate (SE)	12-month Estimate (SE)
Intercept Patient –level						
Gender	−0.006(0.155)	0.068(0.131)	−0.130(0.125)	**−0.869(0.267)**	**−0.549(0.188)**	**−0.517(0.265)**
Age	0.005(0.005)	0.006(0.005)	0.008(0.005)	0.011(0.011)	0.002(0.009)	−0.009(0.009)
BMI	0.010(0.016)	0.018(0.012)	−0.000(0.015)	−0.079(0.033)	−0.012(0.020)	−0.041(0.025)
Smokers	−0.088(0.150)	0.099(0.129)	−0.068(0.135)	**−0.780(0.230)**	**−0.866(0.224**	**−0.630(0.242**
Socio-enconomic	0.043(0.054)	0.075(0.044)	0.045(0.061)	−0.033(0.127)	0.039(0.092)	0.035(0.104)
Education background	0.009(0.101)	−0.041(0.079)	−0.109(0.098)	−0.013(0.210)	−0.061(0.138)	0.091(0.175)
Stress-experience	**−0.290(0.010)**	**−0.268(0.076)**	**−0.389(0.087)**	−0.190(0.183)	−0.154(0.156)	−0.093(0.168)
Number of missing teeth	−0.014(0.029)	0.004(0.024)	−0.021(0.043)	0.015(0.070)	−0.036(0.052)	−0.033(0.819)
% of sites with plaque at baseline	−0.407(0.399)	−0.240(0.308)	−0.137(0.417)	−1.326(0.962)	−0.876(0.821)	−1.302(0.642)
% of sites with BOP at baseline	−0.189(0.440)	−0.005(0.238)	0.068(0429)	1.162(0.740)	0.907(0.615)	0.923(0.008)
% of sites with PD ≥ 5 mm at baseline	**1.43(0.467)**	**2251(0.363)**	**1.468(0.495)**	0.647(0.703)	**1399(0.592)**	1.032(0.743)
Tooth-level						
Tooth position	−0.062(0.035)	**−0.080(0.032)**	−0.047(0.039)	**−0.125(0.049)**	**−0.136(0.039)**	**−0.062(0.045)**
Mobility	**0.211(0.057)**	**0.158(0.048)**	**0.190(0.054)**	**0.115(0.059)**	0.057(0.070)	**0.161(0.071)**
Site-level						
BOP at baseline	**0.607(0.050)**	**0.563(0.051)**	**0.585(0.059)**	**0.542(0.073)**	**0.581(0.062)**	**0.691(0.065)**
Plaque at baseline	0.088(0.045)	0.056(0.048)	0.036(0.048)	−0.009(0.067)	0.078(0.062)	0.064(0.063)
Surface of tooth	**0.254(0.030)**	**0.244(0.031)**	**0.202(0.031)**	**0.470(0.048)**	**0.547(0.045)**	**0.572(0.044)**
Variance						
Subject	0.08	0.04	0.07	0.31	0.21	0.25
Tooth	0.15	0.14	0.16	0.34	0.28	0.30
Site	1.17	1.17	1.33	2.16	2.07	2.06
Total variance % reduction in variance						
Patient	50%	79%	65%	40%	48%	38%
Tooth	25%	22%	20%	3%	7%	3%
Site	7%	6%	6%	4%	8%	10%

boldface representative *P < 0.05*

At the tooth level, more mobility showed significantly greater reduction in PD at 3, 6, and 12 months (*p < 0.05*). Anterior teeth showed a significantly greater reduction in PD only at 6 months (*p < 0.05*). The variables on the tooth level were reduced by 25%, 22%, and 20% of PD reduction at 3, 6, and 12 months when compared with the corresponding Variance Components Models (Table 4).

At the patient level, no stress-experience and more percentage sites with PD ≥ 5 mm at baseline showed significant greater reductions in PD at 3, 6, and 12 months (*p < 0.05*). The variables on the patient level were reduced by 50%, 79%, and 65% of PD reduction at 3, 6, and 12 months when compared with the corresponding Variance Components Models (Table 4).

### The relationship of the risk factors and CAL on healing response

A total of 5724 sites distributed on 1431 teeth in the 54 patients were included in this study. Compared with the baseline, the mean reduction in CAL was 0.66 mm, 0.72 mm, and 0.81 mm at 3, 6, and 12 months.

At the site level, the presence of BOP at baseline and mesiocclusion/distocclusion surface of the tooth showed a significantly greater reduction in CAL at 3, 6, and 12 months (*p < 0.05*). The variables on the site level were reduced by 4%, 8%, and 10% of CAL reduction at 3, 6, and 12 months when compared with the corresponding Variance Components Models (Table 4).

At the tooth level, the anterior teeth compared with the molars showed a significantly greater reduction in CAL at 3, 6, and 12 months (*p < 0.05*). More mobility showed a significantly greater reduction in CAL only at 12 months (*p < 0.05*). The variables for the tooth level were reduced by 3%, 7%, and 3% of CAL reduction at 3, 6, and 12 months when compared with the corresponding Variance Components Models (Table 4).

At the patient level, females and non-smokers showed significantly greater reductions in CAL at 3, 6, and 12 months (*p < 0.05*). More percentage sites with PD ≥ 5 mm at baseline showed a significantly greater reduction in CAL only at 6 months (*p < 0.05*).The variables on the patient level were reduced by 40%, 48%, and 38% of CAL reduction at 3, 6, and 12 months when compared with the corresponding Variance Components Models (Table 4).

## Discussion

The present study used variance component models to correctly specify the periodontal data structure and to compare the risk factors of moderate to severe periodontitis. The results of this study demonstrated that variances at each level were statistically significant, implying that ignoring any level may result in an erroneous finding. The variance components showed that the teeth variable was less similar than the subjects variable, and the sites variable was greater than both, implying that the reduction of PD and CAL is more substantial among different sites. Thus, the subject, tooth, and site levels should be considered when investigating the correlation structure of periodontal data.

A previous study [17] used a “periodontal pocket close” situation as evaluation criteria for the efficacy of periodontal treatment. However, “periodontal pocket close” did not fully reflect the effects of basic treatment, especially for patients with moderate to severe periodontitis. Therefore, in this study we used not only PD reduction but also CAL reduction as indicators to evaluate the efficacy of non-surgical periodontal treatment for patients with severe chronic periodontitis. The results of the present study demonstrated that non-surgical periodontal treatment was significant for moderate to severe periodontitis. Site-level had the most effect on PD and CAL reductions. The result was the same as that in Tu YK’s study [18].

Subject-level showed that males have a higher prevalence of clinical attachment loss. Studies by Hujoel et al. [19] have shown that male attachment loss is ≥3 mm, ≥4 mm, and ≥5 mm higher than 23%, 44%, and 55% that of females, respectively; that males consistently had more attachment loss than females at each age is not surprising due to males’ poor oral health awareness, self-care, and dental visiting practices, which may be related to hormones and oral hygiene habits [20]. Recent studies have shown that even after excluding smoking and oral hygiene, males still have higher morbidity, that relatively severe periodontitis may be related to differences in sex-specific immune function of the X chromosome differences, and that hormone secretion may lead to differences in the immune response of gender [21]. Male and female innate immunity and acquired immunity are quite different: the male body contains more IL-1β and TNF-α than the female body, but for these cytokines, infection and damage are closely linked. Smoking showed a negative impact on CAL reduction, which corroborates data reported in reviews on the effect of smoking on the outcome of periodontal treatment [22]. Smoking seriously affects periodontal health and inhibits osteoblast growth due to aggravated bone resorption. The random intercept models revealed an interaction between smoking and CAL reduction, yielding negative effect of smoking compared with non-smokers [23].Non-surgical periodontal therapy in patients with the poor treatment effect of smoking may inhibit fibroblast cell growth and attachment, affect the repair of periodontal tissue, affect periodontal tissue microcirculation, and impede periodontal tissue metabolism. Similar findings have previously been reported showing that smoking was a significant factor in determining the short-term clinical outcome of non-surgical periodontal treatment [24]. Stress-experience factors included stress, depression, anxiety, and tension. The higher the stress-experience, the higher the level of gingival sulcus fluid IL-6, which may affect immune function [25]. Pressure increases glucocorticoids to suppress the immune response of T cells, which makes it easy to accumulate plaque and affect the effectiveness of the treatment.

The tooth-level covariates continued to show a linear relationship for the percentage of sites with PD ≥ 5 mm at baseline and mobility. The main indicator of the degree of periodontal inflammation is PD. The more percentage of sites with PD ≥ 5 mm at baseline represents a more mobility-described infection and more severe inflammation, which means that then on-surgical periodontal treatment had room for improvement. During the period of observation, the premolars showed less overall change in CAL reduction than the anterior but more than the molars. Molars (M) are considered to have a more doubtful prognosis [26]. The presence of furcation defects, the divergence of roots, the dimensions of the furcation entrances, the concavities on the root surfaces, the cervical enamel projections, and even the more posterior position of the arches are considered factors that influence the establishment and progression of the periodontal destruction of molars. Due to the presence of molar root bifurcation, inflammation is not easy to control, and cleaning the rearward position is difficult. Because of the existence of premolar root surface concaves, infection is not easy to control.

Among the site-level covariates, there were some new discoveries. BOP at baseline and surface of tooth showed positive predictions for PD reduction and CAL reduction with non-surgical periodontal treatment [27]. As we all know, plaque is the initial factor for periodontitis. However, the present study showed that the plaque at baseline and the percentage of sites with plaque at baseline had no significant effect on the prognosis of non-surgical periodontal treatment. Tomasi C et al. [17], in a multilevel approach of factors influencing the outcome of non-surgical periodontal treatment, showed that the aggregated variable of percentage of sites with plaque on the subject-level was not a significant factor, but the presence of plaque at site-level was identified as significant. The main difference is the reason for the research object selected: the Chinese people have relatively poor oral hygiene practices overall, with percentage of sites with plaque at baseline of more than 95%. Therefore, this study did not show an effect of the presence of plaque at the site-level. China is still a developing country; economic development is especially lacking in Northern China, where the socio-economic position and education level is low. The cost of treatment is expensive and not reimbursed, leading to poor awareness of dental initiative, poor oral hygiene, and poor attendance rates in accordance with the development of the treatment plan. Therefore, few patients meet the requirements of the treatment plan.

Taken together, MLM was appropriately applied in the analysis of periodontal data. By means of the relevant data analysis, the risk factors that affected the prognosis of periodontitis could successfully be investigated. Accordingly, the effect of periodontal therapy could be enhanced along with proper control of the relevant risk factors. Although the one-factor evaluation was far from adequate for predicting disease prognosis, multi-factor analysis was overwhelmingly successful in evaluating the prognosis of individual periodontitis [24, 28]. For the reason that various risk factors showed different effects on disease and the same individual was affected by multiple risk factors at the same time, evaluation of the effect on disease prognosis from multiple risk factors on the same individual was particularly important in guiding our clinical work.

## Conclusions

The study found that non-surgical periodontal treatment with regular follow-up visits had a remarkable curative effect for moderate to severe chronic periodontitis in terms of PD and CAL reductions. Periodontal data have been confirmed to have an inherently hierarchical structure. Use of MLM allowed determination of the impact of the patient, the tooth, and the tooth site factors on healing responses to non-surgical periodontal therapy in chronic periodontitis. Through MLM analysis found all three levels had a substantial influence on the reduction of PD and CAL. Site-level had the largest effect on PD and CAL reductions.

## Additional files

Additional file 1: Periodontal risk factors. (DOC 30 kb)

## Abbreviations

BOP: Bleeding on probing
CAL: Clinical attachment loss
MLM: Multilevel modelling
PD: Probing depth
PRA: Proposed periodontal risk assessments
PRC: Periodontal risk calculators

## References

[1] Heitz-Mayfield LJA (2005). Disease progression: identification of high-risk groups and individuals for periodontitis. J Clin Periodontol.

[2] Borrell LN, Papapanou PN (2005). Analytical epidemiology of periodontitis. J Clin Periodontol.

[3] Genco RJ, Borgnakke WS (2013). Risk factors for periodontal disease. Periodontol.

[4] Ramseier CA (2005). Potential impact of subject-based risk factor control on periodontitis. J Clin Periodontol.

[5] Bharti V, Khurana P (2009). Metabolic syndrome and periodontal disease. J Indian Soc Periodontol.

[6] Otomo-Corgel J, Pucher JJ, Rethman MP (2012). State of the science: chronic periodontitis and systemic health. J Evid Based Dent Pract.

[7] Pihlstrom BL (2001). Periodontal risk assessment, diagnosis and treatment planning. Periodontol.

[8] Costa FO, Miranda LO, Pereira EJ (2012). Periodontal risk assessment (Pra) model in a sample of regular and irregular compliers under maintenance therapy: a 3-year prospective study. J Periodontol.

[9] Lindskog S, Blomlöf J, Persson I (2010). Validation of an algorithm for chronicperiodontitis risk assessment and prognostication: risk predictors, explanatory values, measures of quality, and clinical use. J Periodontol.

[10] Peugh JL (2010). A practical guide to multilevel modeling. J Sch Psychol.

[11] Kahn JH (2011). Multilevel modeling: overview and applications to research incounseling psychology. J Couns Psychol.

[12] Albandar JM, Goldstein H (1992). Multi-level statistical models in studies of periodontal diseases. J Periodontol.

[13] Chung H, Beretvas SN (2012). The impact of ignoring multiple membership datastructures in multilevel models. Br J Math Stat Psychol.

[14] Burnside G, Pine CM, Williamson PR (2013). Statistical power of multilevel Modelling in dental caries clinical trials: a simulation study. Caries Res.

[15] Axtelius B, Söderfeldt B, Attström R (1999). A multilevel analysis of factors affecting pocket probing depth in patients responding differently to periodontal treatment. J Clin Periodontol.

[16] Wu X, Offenbacher S, Lopez NJ (2015). Association of interleukin-1 gene variations with moderate to severe chronic periodontitis in multiple ethnicities. J Periodontal Res.

[17] Tomasi C, Leyland AH, Wennström JL (2007). Factors influencing the outcome ofnon-surgical periodontal treatment: a multilevel approach. J Clin Periodontol.

[18] Tu YK, Gilthorpe MS, Griffiths GS (2004). The application of multilevel modeling in the analysis of longitudinal periodontal data--part I: absolute levels of disease. J Periodontol.

[19] Hujoel PP, Loesche WJ, DeRouen TA (1990). Assessment of relationships between site-specific variables. J Periodontol.

[20] Shiau HJ, Reynolds MA (2010). Sex differences in destructive periodontal disease: exploring the biologic basis. J Periodontol.

[21] Tu YK, Gilthorpe MS, Griffiths GS (2004). The application of multilevel modeling in the analysis of longitudinal periodontal data--part II: changes in disease levels over time. J Periodontol.

[22] Omasi C, Leyland AH, Wennström JL (2007). Factors influencing the outcome of non-surgical periodontal treatment: a multilevel approach. J Clin Periodontol.

[23] Zeng J, Williams SM, Fletcher DJ (2014). Reexamining the association between smoking and periodontitis in the dunedin study with an enhanced analytical approach. J Periodontol.

[24] Wan CP, Leung WK, Wong MC (2009). Effects of smoking on healing response to non-surgical periodontal therapy: a multilevel modelling analysis. J Clin Periodontol.

[25] Saini R, Saini S, Saini SR (2010). Periodontitis and psychological stress: a dental view. Ind Psychiatry J.

[26] Heitz-Mayfield L, Trombelli L, Heitz F (2002). A systematic review of the effect of surgical debridement vs. non-surgical debridement for the treatment of chronic periodontitis. J Clin Periodontol.

[27] Rahardjo A, Yoshihara A, Amarasena N (2005). Relationship between bleeding on probing and periodontal disease progression in community-dwelling older adults. J Clin Periodontol.

[28] Clarke P (2008). When can group level clustering be ignored?Multilevel models versus single-level models with sparse data. J Epidemiol Community Health.

