# Supplementary material for: Risk factors of chronic periodontitis on healing response: a multilevel modelling analysis
Source: BMC Med Inform Decis Mak. 2017 Sep 15;17:135. doi: 10.1186/s12911-017-0533-2 (PMC5603071; doi:10.1186/s12911-017-0533-2)
Supplement: Supplementary file 1 — Periodontal risk factors. (DOC 30 kb) [file 12911_2017_533_MOESM1_ESM.doc]

| Periodontal risk factors | | | |
| --- | --- | --- | --- |
| Gender:(Male/Female) |  | Age: |  |
| Stress-experience: (yes/no) |  | BMI:  Education background:（post graduation / graduate / senior school or below） | |
| Smokers:(yes/no) |  | Socio-economic: More than 599.60 USD=1, 299.76-599.60 USD =2,  149.88-299.76 USD=3,  74.94-149.88 USD =4,  less than 74.94 USD=5 | |
